# Drug Screening Boosted by Hyperpolarized Long-Lived States in NMR

**DOI:** 10.1002/cmdc.201402214

**Published:** 2014-09-04

**Authors:** Roberto Buratto, Aurélien Bornet, Jonas Milani, Daniele Mammoli, Basile Vuichoud, Nicola Salvi, Maninder Singh, Aurélien Laguerre, Solène Passemard, Sandrine Gerber-Lemaire, Sami Jannin, Geoffrey Bodenhausen

**Affiliations:** [a] Institut des Sciences et Ingénierie Chimiques, Ecole Polytechnique Fédérale de Lausanne (EPFL)1015 Lausanne (Switzerland) E-mail: aurelien.bornet@epfl.chgeoffrey.bodenhausen@epfl.ch; [b] Department of Biological Chemistry & Molecular Pharmacology, Harvard Medical School240 Longwood Ave., Boston, MA 02115 (USA); [c] Department of Chemistry, Indian Institute of Technology110 016 New Delhi (India); [d] Institut de Chimie Moléculaire de l'Université de Bourgogne (ICMUB), Université de Bourgogne21078 Dijon (France); [e] Bruker BioSpin AGIndustriestrasse 26, 8117 Fällanden (Switzerland); [f] Department of Chemistry, Ecole Normale Supérieure-PSL Research University24 rue Lhomond, 75005 Paris (France); [g] Sorbonne UniversitéUPMC Univ Paris 06, LBM, 4 place Jussieu, 75005 Paris (France); [h] CNRSUMR 7203 LBM, 75005 Paris (France)

**Keywords:** drug discovery, dynamic nuclear polarization, long-lived states, NMR spectroscopy

## Abstract

Transverse and longitudinal relaxation times (*T*_1ρ_ and *T*_1_) have been widely exploited in NMR to probe the binding of ligands and putative drugs to target proteins. We have shown recently that long-lived states (LLS) can be more sensitive to ligand binding. LLS can be excited if the ligand comprises at least two coupled spins. Herein we broaden the scope of ligand screening by LLS to arbitrary ligands by covalent attachment of a functional group, which comprises a pair of coupled protons that are isolated from neighboring magnetic nuclei. The resulting functionalized ligands have longitudinal relaxation times *T*_1_(^1^H) that are sufficiently long to allow the powerful combination of LLS with dissolution dynamic nuclear polarization (D-DNP). Hyperpolarized weak “spy ligands” can be displaced by high-affinity competitors. Hyperpolarized LLS allow one to decrease both protein and ligand concentrations to micromolar levels and to significantly increase sample throughput.

## Introduction

The first step of drug discovery is commonly referred to as *lead identification*. Screening techniques such as enzyme-linked immunosorbent assays (ELISA),[1] surface plasmon resonance (SPR, also known under the trade name Biacore),[2] isothermal titration calorimetry (ITC),[3] fluorescence anisotropy,[4] and an ever-expanding range of nuclear magnetic resonance (NMR) techniques[5] allow one to recognize ligands or fragments thereof in extensive libraries of chemical compounds. The binding of a ligand to a target protein:


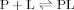
(1)

can be described by a dissociation constant *K*_D_ that gives a measure of the affinity:[6]


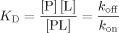
(2)

in which [L], [P], and [PL] are the respective concentrations of the free ligand, the free protein, and the protein–ligand complex, and *k*_on_ and *k*_off_ are second- and first-order rate constants of the association and dissociation reactions. In NMR, a first-order exchange rate is defined as *k*_ex_=(*k*_on_[P]+*k*_off_). If the binding site of the protein is saturated by excess ligand, that is, if [PL]≪[L], one has *k*_ex_≈*k*_off_. Typically, good drugs have small dissociation constants with *K*_D_<1 μm.

Because one can choose from a wide range of observable parameters, NMR spectroscopy offers several methods to study interactions between small ligand molecules and macromolecular targets. It is possible to extract dissociation constants[7] and to obtain structural information about the protein and its complex.[8] Provided the exchange between the free and bound forms of the ligand is faster than the difference of their resonance frequencies,5a, [9] i.e., when *k*_ex_≈*k*_off_≫(π/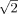)Δ*ν*, where Δ*ν* is the chemical shift difference (in Hz) of the signals in the bound and free states, any observable quantity *ξ*^obs^, be it a frequency or a relaxation rate, is determined by a weighted average of the free and bound forms:[10]


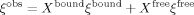
(3)

for which *X*^bound^ and *X*^free^ are the mole fractions of the bound and free ligands. The larger the contrast,


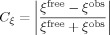
(4)

the more sensitive the frequency or relaxation rate is to ligand–protein binding. This expression is more general than the definition of contrast that we proposed in our first report on the subject.[11]

Several NMR methods based on such a contrast are extensively used nowadays to determine dissociation constants of ligand–protein interactions. The quantity *ξ*^obs^ can be determined by the chemical shifts of one or more selected nuclei of either target proteins[12] or ligands,[13] the translational or rotational diffusion constant of the ligand,[14] the auto-relaxation rates *T*_1_, *T*_2_ and *T*_1ρ_, the rate of magnetization transfer by cross-relaxation (Overhauser effect) between protons belonging to the ligand,[15] the saturation transfer from proteins to ligands determined by difference spectroscopy,[16] or “water-LOGSY” that exploits differences of the rate of transfer of magnetization from bulk water to free or bound ligands by cross-relaxation.[17] Several of these methods rely on differences in rotational correlation times between the free ligand and the protein–ligand complex.[18]

We recently demonstrated that so-called long-lived states (LLS), also known as singlet states (SS) in isolated two-spin systems, can be used very effectively to investigate protein–ligand interactions.[11] Indeed, the protracted lifetimes *T*_LLS_ of these nuclear spin states are exquisitely sensitive to binding to a protein, giving a dramatic contrast between the lifetimes *T*_LLS_ of the bound and free forms. Similar rules apply to the lifetimes *T*_LLC_ of so-called long-lived coherences (LLC).[19] A drawback of both LLS and LLC methods is that the ligands should contain reasonably isolated two-spin systems. We show in this work that it is possible to overcome this limitation by covalent attachment of a “spin-pair label” carrying an isolated two-spin system.

The requirement of rapid exchange underlying Equation (3) implies that “direct” NMR binding experiments cannot be used for ligands with strong affinities (*K*_D_<100 μm), although good drugs typically have much smaller dissociation constants *K*_D_<1 μm. Fortunately, so-called “competition binding” experiments can be used to determine small dissociation constants *K*_D_ of competitors that can displace weak “spy ligands” from the binding sites of target proteins. We show here how a weak ligand with a spin-pair label capable of sustaining an LLS or LLC can be used as a “spy ligand” in competition experiments. If a competitor partly displaces the spy ligand, the lifetimes *T*_LLS_ and *T*_LLC_ of the spy ligand can be dramatically extended. A set of 1D experiments allows one to screen and rank extensive libraries of compounds. Such “indirect” competition binding experiments[20] open the way to the identification of high-affinity ligands with *K*_D_<1 μm, typical of effective drugs. Remarkably, competition binding experiments can also be used to determine poor binding constants *K*_D_>10 mm that are typical of weakly binding “lead compounds” that tend to be difficult to identify in the early stages of drug discovery.[21]

It is clearly desirable to use low concentrations of both proteins and ligands, not only to save expensive materials, but also to avoid protein aggregation and problems with mixtures (“cocktails”) in the manner of combinatorial chemistry. The quest for low ligand concentrations is generally limited by poor sensitivity of NMR. At concentrations [L]<100 μm, NMR spectra with sufficient signal-to-noise ratios require extensive signal averaging. Hyperpolarization of nuclear spins by dissolution dynamic nuclear polarization (D-DNP)[22] can overcome this problem. By microwave irradiation of samples at temperatures close to *T*=1.2 K, the polarization of electron spins can be transferred to protons or other nuclei, followed by rapid dissolution of the hyperpolarized samples and their transfer to a high-resolution NMR spectrometer for detection. The technique has not been very popular for ^1^H and ^19^F nuclei so far, because rapid *T*_1_ relaxation tends to cause loss of polarization during the transfer from the polarizer to the spectrometer. Enhancements *ε*_DNP_ up to five orders of magnitude can be obtained for nuclei with low gyromagnetic ratios, while enhancements 100<*ε*_DNP_<1000 can be achieved for ^1^H or ^19^F nuclei.[23] Spin-pair-labeled molecules containing isolated spins designed for LLS and LLC experiments also feature fairly long *T*_1_ values which also makes them suitable for dissolution DNP.

### Long-lived states experiments

**Figure 1:**
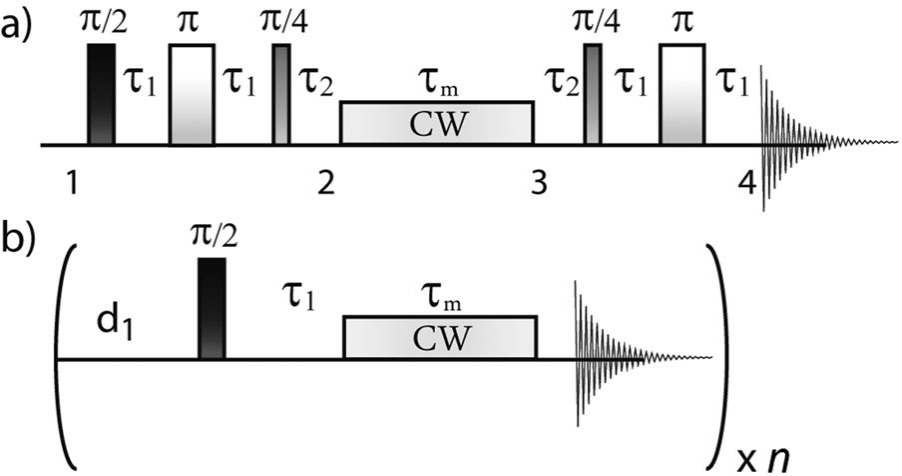
Experiments for long-lived states (LLS) and long-lived coherences (LLC). a) Pulse sequence used to excite, sustain, and observe LLS.[25] An *rf* field is applied between time points 2 and 3 with the carrier halfway between the chemical shifts of the two spins in order to make them effectively equivalent. The conversion is most efficient if *τ*_1_=1/4 *J*_IS_ and *τ*_2_=1/2Δ*ν*_IS_. b) Pulse sequence designed to excite, sustain, and detect LLC. Typically, *τ*_1_=1/2Δ*ν*_IS_ to achieve an efficient conversion of *I*_y_+*S*_y_ into *I*_x_−*S*_x_, and d_1_=5*T*_1_. A variant for single-scan LLC spectroscopy is described in the Supporting Information.

Long-lived states (LLS), first described by Levitt and co-workers,[24] have the unique property that their populations relax with time constants that can be much longer than longitudinal relaxation time constants (*T*_LLS_≫*T*_1_). For pairs of protons, *T*_LLS_/*T*_1_ ratios as large as 60 have been observed in R–CH=CH–R’ systems. To perform an LLS experiment, one has to: 1) Start with a system comprising two nonequivalent spins and convert their populations into a density operator corresponding to a singlet–triplet imbalance (Figure 1 a, 1–2);24a, [25] 2) Sustain the LLS by suppressing the effects of the chemical shift difference (Figure 1 a, 2–3), usually by applying a resonant radiofrequency (*rf*) field during a sustaining time *τ*_m_, with the carrier (*ν*_rf_) placed halfway between the chemical shifts of the two spins;24a,d, [26] LLS are efficiently sustained by an *rf* field that is at least five times larger than the chemical shift difference between the two spins. 3) After turning off the resonant *rf* field, a suitable pulse sequence can convert the singlet–triplet imbalance back to observable magnetization (Figure 1 a, 3–4). The lifetime of the LLS can be determined by fitting the signal intensities recorded as a function of *τ*_m_ to the exponential function exp(−*τ*_m_/*T*_LLS_).

On the other hand, long-lived coherences (LLC)[19] have the property that they relax with time constants that can be much longer than *transverse* relaxation time constants (*T*_LLC_≫*T*_2_). The principles of LLC spectroscopy are briefly reviewed in the Supporting Information.

## Results and Discussion

### Enhanced contrast of LLS

In direct titration experiments, i.e., in the absence of competitors, the relaxation rate of a (weak or intermediate) spy ligand is measured by titration as a function of the protein-to-ligand ratio. An LLS associated with a ligand L bound to a target protein P will relax faster, that is, *T*_LLS_^bound^≪*T*_LLS_^free^. The relaxation properties of free and bound ligands contribute to increase the contrast *C*(*T*_LLS_) of the method. According to Equation (4), one may define the contrast as:


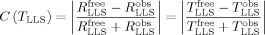
(5)

where *R*_LLS_^obs^ and *T*_LLS_^obs^ are the averaged parameters observed for the rapid equilibrium between free and bound forms of the ligand in the sense of Equation (3), while *R*_LLS_^free^ and *T*_LLS_^free^ refer to the free ligand in the absence of protein. By analogy, an expression for the contrast *C*(*T*_LLC_) of long-lived coherences can be derived from Equation (5) by replacing LLS with LLC.

**Figure 2:**
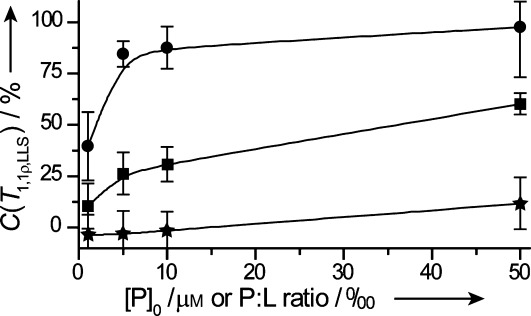
Contrast of life-times *T*_LLS_, *T*_1_, and *T*_1ρ_ of ligands binding to proteins. Experimental contrast for *T*_LLS_ (•), non-selective *T*_1ρ_ (▪), and non-selective *T*_1_ (★) methods for the diastereotopic pair of protons on the middle glycine residue of the tripeptide GGR in a solution with a fixed concentration [L]=1 mm and a variable trypsin concentration 0.5 μm<[P]_0_<50 μm in D_2_O at 8 °C at 11.7 T (500 MHz for protons).

To demonstrate the enhancement of the contrast *C*(*T*_LLS_) with respect to the contrast *C*(*T*_1_) and *C*(*T*_1ρ_), binding experiments were carried out for a 1 mm solution of the tripeptide ligand glycine-glycine-arginine (GGR) in the presence of its protein target trypsin in the range 0.5<[P]<50 μm, using various methods (*T*_LLS_, nonselective *T*_1_ and nonselective *T*_1ρ_). Figure 2 shows that the LLS method can work with a protein–ligand ratio that is ∼25-fold lower than required for the well-known nonselective *T*_1ρ_ method, whereas the nonselective *T*_1_ contrast remains below *C*(*T*_1_)<10 % even at the highest protein concentration [P]=50 μm.

### Spin-pair labeling

A drawback of screening by LLS or LLC is that the ligands must carry a pair of nonequivalent spins −1/2. We therefore developed a synthetic labeling strategy comprising two steps: 1) the identification of a “spy ligand” that binds weakly to the target protein, and 2) the functionalization (see Supporting Information) of this ligand by attaching a “spin-pair label” that can carry LLS or LLC. By way of illustration, 3-bromothiophene-2-carboxylic acid (“BT”), which is known to have long lifetimes *T*_LLS_ and *T*_LLC_,[27] was covalently attached to the tripeptide GGR, a weak binder for trypsin. The resulting spin-pair-labeled tripeptide is henceforth called BT-GGR.

**Figure 3:**
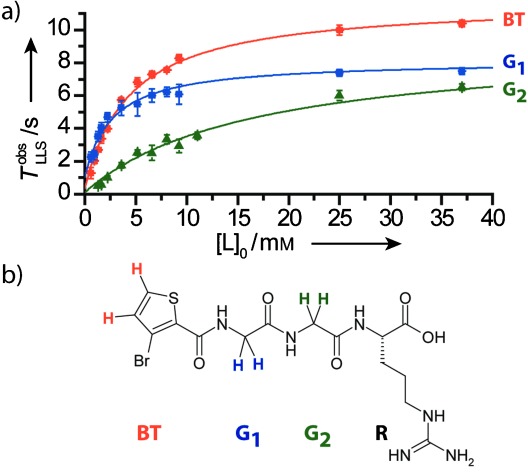
LLS titration experiments. a) Observed LLS lifetimes of the three proton pairs on the spin-pair-labeled tripeptide BT-GGR as a function of the ligand concentration, in the presence of 25 μm trypsin in D_2_O at 25 °C and 11.7 T (500 MHz for protons). b) Pairs of protons capable of sustaining LLS in BT-GGR: on bromothiophene BT (orange), on the N-terminal glycine G_1_ (blue) and on central glycine G_2_ (green) which is close to the arginine residue that binds to the protein.

Despite some steric effects and long-range dipolar relaxation mechanisms in the spin-pair-labeled tripeptide BT-GGR, the two aromatic protons of the bromothiophene group retain a remarkably long lifetime *T*_LLS_^free^(BT)=11.7±0.7 s. In this particular peptide, the diastereotopic pairs of α-protons on the two glycine residues of BT-G_1_G_2_R can also be used to excite LLS and have lifetimes *T*_LLS_^free^(G_1_)=10.4±0.5 s and *T*_LLS_^free^(G_2_)=9.3±0.5 s (see Figure 3 b).

The observed averaged relaxation rate of LLS in the presence of a protein can be derived from Equation (3), using the definition of *K*_D_ of Equation (2) and assuming saturation, i.e., [L]_0_≫[PL], so that [L]_0_−[PL]≈[L]_0_:


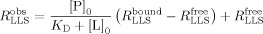
(6)

If one measures *R*_LLS_^obs^ as a function of the concentration [L]_0_ of a ligand while [P]_0_ is kept constant, it is possible to determine *K*_D_ by fitting to Equation (6). The spin-pair-labeled spy ligand L=BT-GGR was added to a solution of [P]_0_=25 μm trypsin over a range 0.5<[L]_0_<40 mm. At each concentration [L]_0_, the observed relaxation times *T*_LLS_^obs^=1/*R*_LLS_^obs^ of three different pairs of protons (belonging to the bromothiophene group and to the middle and the terminal glycines) were measured using the pulse sequence of Figure 1. Figure 3 a shows how the titration curves can be fitted to Equation (6). As expected, nearly the same dissociation constants were obtained for the three proton pairs that can sustain LLS in BT-GGR: *K*_D_(BT)=0.18±0.03 mm, *K*_D_(G_1_)=0.24±0.01 mm, *K*_D_(G_2_)=0.21±0.02 mm. The LLS fitted lifetime of G_2_ (i.e., the glycine closest to the arginine) in the bound form (*T*_LLS_^bound^(G_2_)=16±1 ms) is shorter than for the two other LLS sites (*T*_LLS_^bound^(BT)=90±20 ms, *T*_LLS_^bound^(G_1_)=110±40 ms). This shorter *T*_LLS_^bound^ is believed to be due to the fact that the arginine, and thus also the glycine G_2_, enter more deeply into the active site of trypsin.

### Competition binding experiments

Once a weak ligand has been identified and characterized by titration, it can be used as a “spy ligand” in competition experiments.[20] When stronger ligands are added, the lifetime *T*_LLS_^obs^ of the spy ligand give information about the dissociation constant *K*_D_^strong^ of the competitor. Note that the competitor need not contain any spin pairs that can sustain an LLS or LLC. Moreover, as the changes in *T*_LLS_^obs^ need only be observed for the weak ligand, there are no requirements for the stronger ligands to fulfill the fast-exchange condition. This implies that the accessible range of dissociation constants *K*_D_^strong^ of the competitor can lie in a range 0.1 nm<*K*_D_^strong^<100 nm. When *K*_D_^strong^<0.1 nm, one can detect a large effect on the lifetime *T*_LLS_^obs^ of the weak spy ligand, but it is not possible to rank the ligands according to their affinities. As the strong competitors themselves need not be observed directly, their concentration can also be lowered, typically to the same level as the concentration of the protein, that is, to [L]_0_≈[P]_0_, which may typically be in the single-digit micromolar range.

When a stronger competitor blocks the active site of the protein, the weak spy ligand will no longer have free access to its target. The concentration [P]_free_ of the protein that remains free to bind the weak ligand can be derived from the definition of the dissociation constant *K*_D_^strong^ of the competitor:


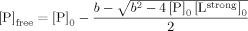
(7)

where *b*=([P]_0_+[L^strong^]_0_+*K*_D_^strong^) and [L^strong^]_0_ is the total concentration of the competitor. As the amount of free available protein decreases, the effects of the protein on the lifetime *T*_LLS_^obs^ of the weak spy ligand will be less pronounced. Here, the approximation that the binding sites are saturated by ligands cannot be made, since [L^strong^]_0_≈[P]_0_. To describe the relaxation rate *R*_LLS_^obs^ of the weak spy ligand in competition experiments, [P]_0_ in Equation (6) must be replaced by [P]_free_ of Equation (7).

**Figure 4:**
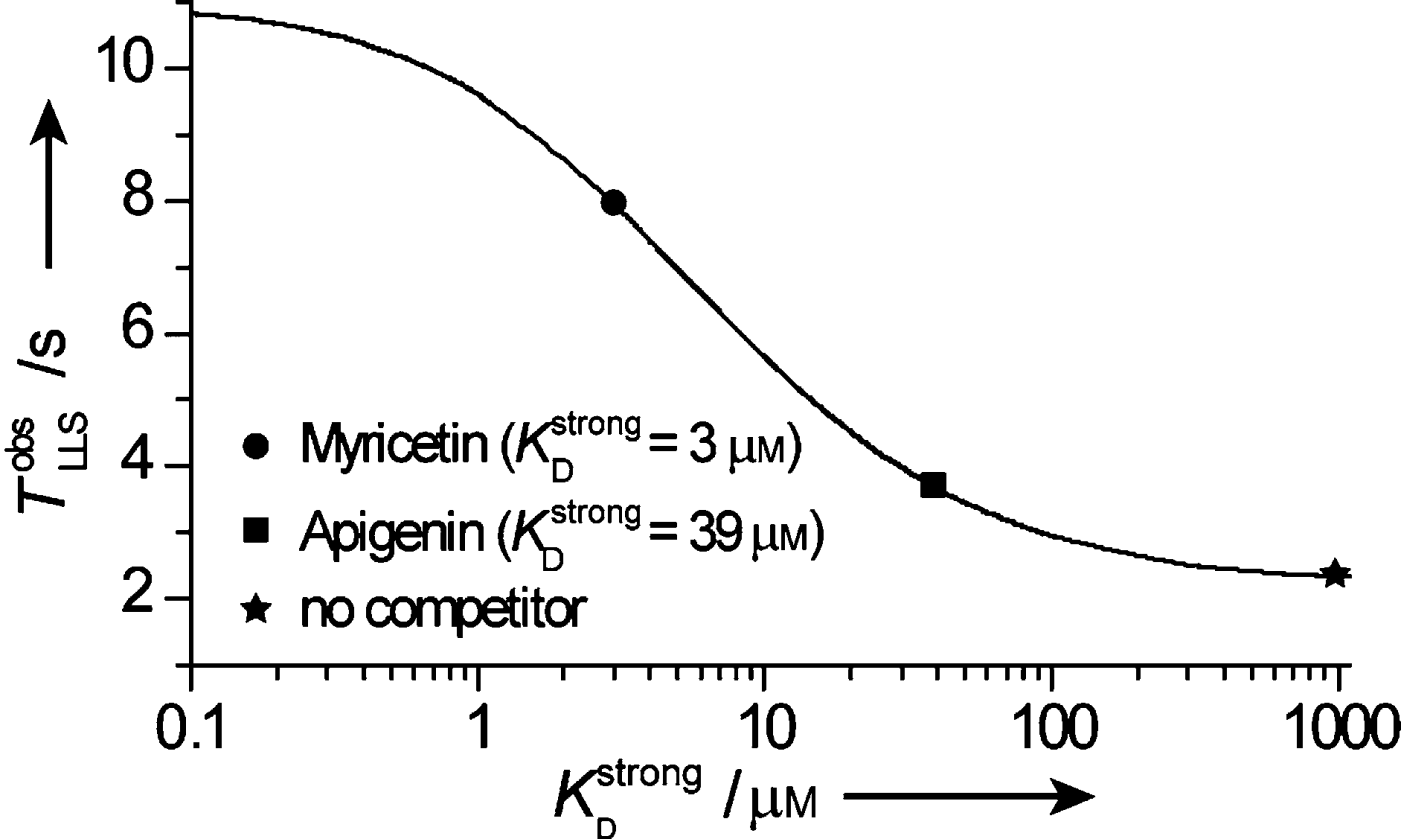
Influence of a competitor on the LLS lifetime of a weak ligand. The LLS lifetimes *T*_LLS_^obs^ of the pair of aromatic protons of the bromothiophene spin-pair label as a function of the dissociation constant of a competing stronger ligand, calculated using Equations (6) and (7). The parameters of the weak ligand BT-GGR were obtained from the fit of the data in Figure 3: *K*_D_=0.2 mm, *T*_LLS_^bound^=0.1 s, *T*_LLS_^free^=11 s, [L]_0_=0.5 mm, [P]_0_=25 μm, and [*L*^s^]_0_=50 μm. The three points correspond to *T*_LLS_^obs^ in the presence of myricetin (*K*_D_^strong^=3 μm, •), apigenin (*K*_D_^strong^=39 μm, ▪) and in the absence of any competitor (★) calculated for these conditions.

Once the dissociation constant *K*_D_^weak^ of the weak spy ligand and its LLS lifetime in the bound form *T*_LLS_^bound^ are known, it is possible to optimize [L^strong^]_0_ and [P]_0_ to rank strong competitors according to their binding strengths. Figure 4 shows the calculated *T*_LLS_^obs^(BT) of the bromothiophene protons in BT-GGR if [P]_0_=25 μm and [L^strong^]_0_=50 μm as function of *K*_D_^strong^. Under these conditions, *T*_LLS_^obs^ changes dramatically between *K*_D_^strong^=100 μm and *K*_D_^strong^=1 μm.

**Figure 5:**
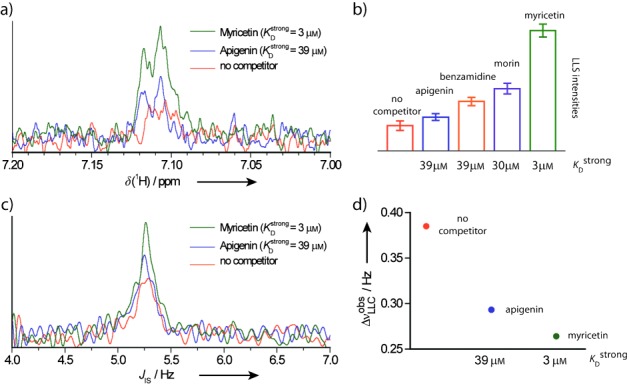
LLS and LLC competition binding experiments. a) Signals of one of the two aromatic protons of bromothiophene of 0.5 mm of the weak spy ligand BT-GGR in the presence of 25 μm trypsin, using the LLS sequence of Figure 1 with a sustaining time *τ*_m_=3 s in D_2_O at 25 °C and 11.7 T (500 MHz for protons) 1) in the absence of any competitor (orange), 2) in competition with 50 μm of the intermediate ligand apigenin (blue), and 3) in competition with 50 μm of the stronger ligand myricetin (green). b) Peak intensities of one of the aromatic protons of BT-GGR under the same conditions as in panel a), without competitor or in the presence of apigenin, benzamidine, morin, or myricetin. The better the binding, the smaller the dissociation constant, and the more intense the LLS signal of the displaced spin-pair-labeled spy ligand BT-GGR. c) LLC spectra of the two aromatic protons of the bromothiophene group of BT-GGR acquired with the “on-the-fly” sequence shown in Figure S2b of the Supporting Information, under the same conditions as in panel a). The stronger the binding of competitors, the greater the displacement of the spy ligand BT-GGR, the narrower the peaks in its LLC spectra, and the more intense the signals. d) Line widths of LLC peaks [Δ*ν*_LLC_^obs^=1/(π*T*_LLC_^obs^)].

A library of competing ligands can thus be ranked according to their affinities by observing the LLS signal of the weak spy ligand. Under the conditions shown in Figure 4, one can easily rank competing ligands with great accuracy provided 1 μm<*K*_D_^strong^<100 μm. Note that the LLS sequence of Figure 1 can be used with a single sustaining delay *τ*_m_. This strategy is compatible with dissolution DNP, as discussed below. As *T*_LLS_^obs^ of the weak spy ligand is longer in the presence of a stronger competitor, the LLS signal intensity of the spy ligand after a suitably chosen delay *τ*_m_ will be higher. LLS spectra with *τ*_m_=3 s were recorded with 0.5 mm BT-GGR, in the presence of [P]_0_=25 μm trypsin with four different competitors, all with [L^strong^]_0_=50 μm: myricetin (*K*_D_^strong^=3 μm), morin (*K*_D_^strong^=30 μm), apigenin (*K*_D_^strong^=39 μm)[28] and benzamidine (*K*_D_^strong^=39 μm).[29] Figure 5 a shows three of these five LLS spectra, obtained either without competitor, with apigenin, or with myricetin. Figure 5 b shows the signal intensities of the weak spy ligand BT-GGR in the presence of one of the four competing ligands. The same kind of information can be derived from the lifetimes of long-lived coherences (Figure 5 c,d).

### Hyperpolarization by dissolution DNP

Ligands with covalently attached spin-pair labels such as BT-GGR contain spins with long *T*_1_ values and are therefore suitable for hyperpolarization by dissolution DNP. Provided *T*_1_(^1^H)>1 s, a sufficient fraction of the hyperpolarized magnetization can be preserved during transfer from the DNP polarizer to the NMR spectrometer.

In a glass-forming solvent mixture H_2_O/D_2_O/[D_6_]DMSO (*v*/*v*/*v*=5:35:60), 10 mm BT-GGR was dissolved with 25 mm 4-hydroxy-2,2,6,6-tetramethylpiperidine-1-oxyl (TEMPOL). Five frozen beads (50 μL) of this solution were loaded together with five frozen beads (50 μL) of 3 m ascorbate[30] into a home-built DNP polarizer[31] operating at *B*_0_=6.7 T and *T*=1.2 K. The sample was irradiated with microwaves at a frequency *f*_μW_=188.3 GHz and power *P*_μW_=100 mW. Unlike trityl radicals, the nitroxyl radical TEMPO is an efficient polarizing agent for ^1^H spins, because it has a broad ESR line Δ*ω*_ESR_>*ω*_0_(^1^H).[32] At *B*_0_=6.7 T, a proton polarization up to *P*(^1^H)=90 % can be obtained,31b while *P*(^1^H) is only ∼40 % in polarizers operating at *B*_0_=3.35 T. After ∼15 min of microwave irradiation, a steady-state proton polarization *P*(^1^H) is reached. The DNP sample can be rapidly dissolved in 0.7 s with 5 mL of hot D_2_O (*P*=1 MPa, *T*=400 K) and transferred to a 11.7 T NMR spectrometer in 4.5 s through a “magnetic tunnel” so that *B*_0_>0.8 T during transfer, which is particularly important to preserve the polarization of ^1^H and ^19^F nuclei.[33] A fraction (400 μL) of the hyperpolarized solution is then injected in ∼2 s into a 5 mm NMR tube containing 250 μL D_2_O and, depending on the conditions, 3.65 μm trypsin and 3.65 μm of a competitor such as myricetin. After injection, the final solution has a concentration of 1.4 μm protein, 1.4 μm competitor, and 120 μm hyperpolarized spy ligand BT-GGR. After a 3 s interval to allow proper mixing, a reference free induction decay is observed in 0.5 s after exciting transverse magnetization with a single 5° pulse to control the quality of the hyperpolarized sample and to normalize the signal intensity of the spy ligand to its known concentration. This is immediately followed by an LLS sequence as described in Figure 1 with a fixed sustaining time *τ*_m_=3 s.

The DNP enhancements of the aromatic protons of the spin-pair-labeled spy ligand BT-GGR were on the order of *ε*_DNP_=100–200, relative to Boltzmann equilibrium at 25 °C and 11.7 T (500 MHz for protons). A significant fraction of the proton hyperpolarization was lost during the 10 s interval between dissolution and signal acquisition, but a faster sample injection device[33] could decrease this interval to 1.2 s.

**Figure 6:**
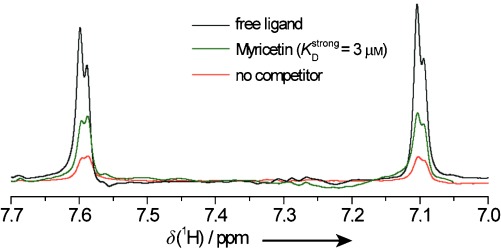
DNP-enhanced LLS competition binding experiments. DNP-enhanced LLS spectra of the two aromatic protons of bromothiophene in BT-GGR 120 μm after a sustaining time *τ*_m_=3 s, 1) without protein (black), 2) in the presence of 1.4 μm trypsin (orange), 3) with 1.4 μm trypsin and 1.4 μm myricetin as competitor (green). All spectra were acquired in a single scan in D_2_O, at 25 °C and 11.7 T (500 MHz for protons).

Figure 6 shows DNP-enhanced LLS spectra of 1) 120 μm of the spin-pair-labeled spy ligand BT-GGR in the absence of protein, 2) the same upon addition of 1.4 μm trypsin, and 3) the same with further addition of 1.4 μm myricetin as competitor. A dramatic decrease of the LLS signal intensity stemming from BT-GGR is observed upon adding trypsin. The contrast defined in Equation (5) is *C*(*T*_LLS_)=75 %. Addition of an equimolar amount of the competitor myricetin leads to a partial displacement of the spy ligand that can be readily detected by the revival of its LLS signal. With only 120 μm of the spin-pair-labeled spy ligand BT-GGR, the DNP-enhanced LLS spectrum of Figure 6 recorded in a single scan after *τ*_m_=3 s has a signal-to-noise ratio (SNR) of 130. Under the same conditions, but without DNP, an accumulation of 225 transients for ∼1 h was necessary to reach the same SNR. A DNP-enhanced LLS spectrum of BT-GGR with a concentration as low as 10 μm could be recorded with an SNR of 16. Clearly, DNP allows one to decrease the concentration of ligands, but the protein concentration should not be further decreased. In fact, according to Equation (6), the contrast *C*_LLS_ would decrease if the limit *K*_D_+[L_0_]≈*K*_D_ were to be reached. Without DNP, using a 50-fold increase in ligand concentration (Figure 5), 256 transients had to be accumulated in 100 min to obtain an SNR of 8. The experimental conditions can be adapted depending on the primary objective: low concentrations of either protein or ligand, rapid throughput, high sensitivity for the displacement by a competitor or high SNR. In Figure 6, the conditions were optimized for high SNR and high contrast upon addition of a competitor, albeit at the expense of a slightly higher ligand concentration and longer polarization buildup time. To attain faster throughput, one could polarize at a higher temperature *T*=4.2 K and *B*_0_=6.7 T, where proton polarization *P*(^1^H)=25 % can be reached by DNP in ∼2 min.31b The price to pay would be an approximate threefold lower SNR. Similarly, at *T*=1.2 K and *B*_0_=3.35 T, as in commercially available DNP polarizers, *P*(^1^H)=40 % can be reached in ∼6 min.[34]

## Conclusions

The covalent attachment of “spin-pair labels” such as bromothiophene (BT) permits one to broaden the scope of the LLS and LLC screening methods to virtually any weak ligand in the fast exchange regime. By way of example, bromothiophene carboxylic acid was attached to the N terminus of a tripeptide, but other “spin-pair labels” could be designed. Spy ligands with higher sensitivity to binding could be engineered with the following features: 1) spy ligands with enhanced *T*_LLS_^free^, 2) LLS functionalizing groups closer to the binding site, 3) the use of nearly equivalent spins.[35] Our labels are far less bulky than chromophores used in fluorescence experiments, and should not induce significant steric impediments to binding. Relative to other NMR screening methods, LLS and LLC offer much improved contrast. For the same ligand concentration (i.e., for the same experimental time), the protein concentrations can be greatly decreased, giving access to poorly soluble protein targets and decreasing the risk of aggregation. When used in competition mode, both LLS and LLC methods allow one to rank high-affinity ligands using simple 1D experiments. Because there are few protons in the spin-pair label, they tend to have long *T*_1_ relaxation times, so that drug screening experiments using LLS or LLC can be, in principle, readily coupled to D-DNP. Currently, access to this technology remains limited to a few specialized groups, but a dedicated LLS-DNP screening apparatus could be made commercially available in the near future. As demonstrated below, this approach allows one to decrease the concentrations of spy ligands, competitors and target proteins. In our current setup, the time required for transfer from the polarizer to the detection magnet is similar to *T*_1_(^1^H) of the spy ligand. An acceleration of the transfer would benefit the remaining proton polarization and thus the SNR. Such improvements would allow either a further decrease in ligand concentration or an increase in sample throughput.

## Experimental Section

**Samples**: BT-GGR (see Supporting Information for details about the synthesis) was titrated over the range 0.5<[L]_0_<40 mm into 25 μm type IX-S trypsin from porcine pancreas (Sigma–Aldrich). As internal concentration standard, 5 mm *tert*-butyl alcohol (>99.5 %, Sigma–Aldrich) was added. Five solutions containing 0.5 mm BT-GGR and 25 μm trypsin in D_2_O were prepared for competition experiments: 1) without competitor, 2) with 50 μm myricetin (>96 %, Sigma–Aldrich), 3) with 50 μm morin hydrate (Sigma–Aldrich), 4) with 50 μm apigenin (>97 %, Sigma–Aldrich), and 5) with 50 μm benzamidine hydrochloride (>99 %, Sigma–Aldrich).

**Experimental procedures**: During the titrations, 2 μL aliquots of 150 mm BT-GGR were added to 300 μL of the starting solution. The lifetimes *T*_LLS_^obs^ were determined by single-exponential fitting of signal intensities observed with the LLS sequence of Figure 1, setting the sustaining field strength *B*_1_=5Δν_IS_, and using N=10 different delays *τ*_m_=*n*_1_Δ*τ*_m_ with Δ*τ*_m_=*T*_LLS_^expected^/10 and *n*_1_=1, 3, 6, 10, 15, 21, 29, 37, 46, 56. All measurements were performed at 25 °C on a 500 MHz (11.7 T) Avance Bruker spectrometer equipped with an inverse detection 5 mm CryoProbe.

**Dissolution-DNP experiments**: Solutions of 10 mm BT-GGR in the glass-forming mixture H_2_O/D_2_O/[D_6_]DMSO (*v*/*v*/*v*=5:35:60) were doped with 25 mm TEMPOL (Sigma–Aldrich). Five frozen beads of 10 μL each of this mixture were inserted with five frozen beads of 10 μL each containing 3 m ascorbate in D_2_O to scavenge the radicals after dissolution. DNP was performed at 1.2 K and 6.7 T in a home-built polarizer by applying CW microwave irradiation at *f*_μW_=188.3 GHz and *P*_μW_=100 mW. The beads were dissolved together in 0.7 s with 5 mL D_2_O, preheated at *T*=400 K at *P*=1.0 MPa, and transferred in 4.5 s by pressurizing with helium gas at 0.6 MPa to an 11.7 T Bruker magnet via a 1.5 mm inner-diameter PTFE tube running through a magnetic tunnel of 5 m length. After injection in 2 s into NMR tubes containing 250 μL of D_2_O to allow field-frequency locking before and during injection, plus 1) 250 μL of 3.65 μm trypsin, or 2) 250 μL of 3.65 μm trypsin and 3.65 μm myricetin. A 5° detection pulse was applied to record a hyperpolarized ^1^H signal for reference, followed by a single LLS sequence with a sustaining delay *τ*_m_=3 s.

## Supporting Information

As a service to our authors and readers, this journal provides supporting information supplied by the authors. Such materials are peer reviewed and may be re-organized for online delivery, but are not copy-edited or typeset. Technical support issues arising from supporting information (other than missing files) should be addressed to the authors.

miscellaneous_information
