# Supplementary material for: Drug Screening Boosted by Hyperpolarized Long-Lived States in NMR
Source: ChemMedChem. 2014 Sep 4;9(11):2509–15. doi: 10.1002/cmdc.201402214 (PMC4506523; doi:10.1002/cmdc.201402214)
Supplement: Supplementary file 1 — miscellaneous_information [file cmdc0009-2509-sd1.pdf]

CHEMMEDCHEM
Chemistry & Drug Discovery

Supporting Information

© Copyright Wiley-VCH Verlag GmbH & Co. KGaA, 69451 Weinheim, 2014

Drug Screening Boosted by Hyperpolarized Long-Lived States in NMR

Roberto Buratto,[a] Aurélien Bornet,*[a] Jonas Milani,[a] Daniele Mammoli,[a] Basile Vuichoud,[a] Nicola Salvi,[a, b] Maninder Singh,[a, c] Aurélien Laguerre,[a, d] Solène Passemard,[a] Sandrine Gerber-Lemaire,[a] Sami Jannin,[a, e] and Geoffrey Bodenhausen*[a, f, g, h]

cmdc_201402214_sm_miscellaneous_information.pdf

Synthesis of a spin-pair-labeled tripeptide

Abbreviations:

ACN	Acetonitrile
Anal. Calc.	Analysis calculated
Atm	Atmosphere
DCM	Dichloromethane
DIPEA	N, N'-Diisopropylethylamine
DMF	N, N-Dimethylformamide
DMSO	Dimethyl sulfoxide
Equiv	Equivalent
ESI	Electrospray ionization
Fmoc	Fluorenylmethyloxycarbonyl chloride
HOBt	Hydroxybenzotriazole
HPLC	High-performance liquid chromatography
hr	Hour
HRMS	High resolution mass spectrometry
IR	Infrared
LC-MS	Liquid chromatography - mass spectrometry
MeOH	Methanol
min	Minute
NMR	Nuclear magnetic resonance
Pbf	2,2,4,6,7-pentamethyldihydrobenzofuran-5-sulfonyl
rpm	Revolutions per minute
rt	Room temperature
SPPS	Solid phase peptide synthesis
SN1	Nucleophilic substitution 1
TBTU	O-(Benzotriazol-1-yl)-N,N,N',N'-tetramethyluronium tetrafluoroborate
TFA	Trifluoroacetic acid
TIS	Triisopropylsilane
t_R	Retention time

I- Synthesis

The synthesis of the spin-pair-labeled tripeptide 3-bromothiophene-2-carboxamido-Gly-Gly-Arg (henceforth ‘BT-GGR’) was performed by solid phase peptide synthesis (SPPS) using 2-chlorotrityl chloride resin and Fmoc protected amino acids (**Scheme S1**). The first step of this synthesis is a SN_1 substitution of Fmoc-Arg(Pbf)-OH on the resin. All remaining reactive 2-chlorotrityl groups were then capped with MeOH. The tripeptide was elaborated by successive couplings of Fmoc protected amino acids in the presence of HOBt and TBTU followed by deprotection of the N-terminus under basic conditions. Finally, 3-bromothiophene-2-carboxylic acid was conjugated at the N-terminus of the tripeptide. Cleavage from the resin, followed by deprotection of the arginine side chain, afforded the spin-pair-labeled tripeptide **6,** which was purified by HPLC.

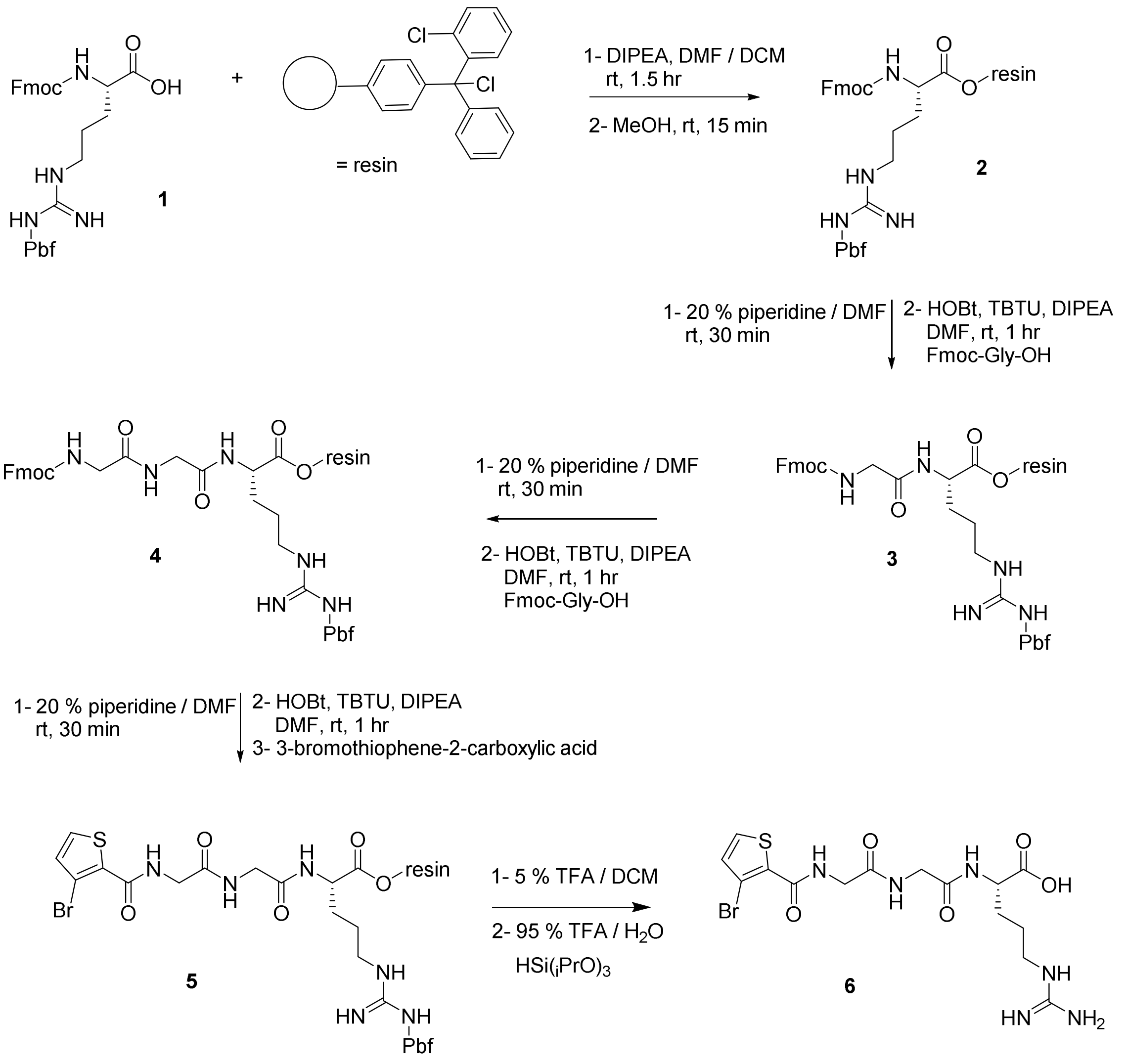

Scheme S1: Synthesis of spin-pair-labeled linear tripeptide BT-GGR **6**.

II- Synthetic steps

N-Fmoc-Arg(Pbf)-O-resin (2): After swelling with dry DCM (80 mL) for 30 min, the 2-chlorotrityl chloride resin (0.83 mmol.g^{-1}, 1 equiv, 2.49 mmol, 3 g) was treated with a solution of Fmoc-Arg(Pbf)-OH (**1**) (2 equiv, 4.98 mmol, 3.23 g) in dry DCM/dry DMF (80 mL/10 mL) and DIPEA (8 equiv, 19.92 mmol, 2.58 g, 3.52 mL) at rt for 1.5 hr agitated at 125 rpm. The reaction was performed in a 400 mL filtration tube with a polyethylene frit. The resin was washed with DMF (3 × 100 mL) and DCM (3 × 100 mL) and dried for 12 hr *in vacuo* to give the N-Fmoc-Gly-O-resin (**2**). MeOH (30 mL) was added to cap the free sites, and the reaction mixture was shaken for 15 min at 125 rpm. The resin was washed with DMF (3 × 100 mL) and DCM (3 × 100 mL).

N-Fmoc-Gly-Arg(Pbf)-O-resin (3): The Fmoc-protected resin (**2**) was suspended in a solution of 20% piperidine in DMF (30 mL) for 30 min and agitated at 125 rpm to give the N-deprotected resin. The resin was washed with DMF (3 × 100 mL), DCM (3 × 100 mL), and DMF (1 × 100 mL). Fmoc-Gly-OH (1.5 equiv, 3.73 mmol, 1.11 g), HOBt (3 equiv, 7.47 mmol, 1.01 g), TBTU (3 equiv, 7.47 mmol, 2.40 g)

and DIPEA (6 equiv, 14.94 mmol, 1.94 g, 2.63 mL) were dissolved in dry DMF (30 mL). The solution was added to the N-deprotected resin. The reaction mixture was shaken at rt for 1 hr at 125 rpm to give Fmoc-Gly-Arg(Pbf)-O-resin (**3**) which was washed with DMF (3 × 100 mL), DCM (3 × 100 mL), DMF (1 × 100 mL).

N-Fmoc-Gly-Gly-Arg(Pbf)-O-resin (4): The Fmoc-protected resin (**3**) was suspended in a solution of 20% piperidine in DMF (30 mL) for 30 min and agitated at 125 rpm to give the N-deprotected resin. The resin was washed with DMF (3 × 100 mL), DCM (3 × 100 mL), and DMF (1 × 100 mL). Fmoc-Gly-OH (1.5 equiv, 3.73 mmol, 1.11 g), HOBt (3 equiv, 7.47 mmol, 1.01 g), TBTU (3 equiv, 7.47 mmol, 2.40 g), and DIPEA (6 equiv, 14.94 mmol, 1.94 g, 2.63 mL) were dissolved in dry DMF (30 mL). The solution was added to the N-deprotected resin. The reaction mixture was shaken at rt for 1 hr at 125 rpm to give Fmoc-Gly-Gly-Arg(Pbf)-O-resin (**4**) which was washed with DMF (3 × 100 mL), DCM (3 × 100 mL), and DMF (1 × 100 mL).

3-bromothiophene-2-carboxamido-Gly-Gly-Arg(Pbf)-O-resin (5): The Fmoc-protected resin (**4**) was suspended in a solution of 20% piperidine in DMF (30 mL) for 30 min and agitated at 125 rpm to give the N-deprotected resin. The resin was washed with DMF (3 × 100 mL), DCM (3 × 100 mL), and DMF (1 × 100 mL). 3-bromothiophene-2-carboxylic acid (1.5 equiv, 3.73 mmol, 0.77 g), HOBt (3 equiv, 7.47 mmol, 1.01 g), TBTU (3 equiv, 7.47 mmol, 2.40 g), and DIPEA (6 equiv, 14.94 mmol, 1.94 g, 2.63 mL) were dissolved in dry DMF (30 mL). The solution was added to the N-deprotected resin. The reaction mixture was shaken at rt for 1 hr at 125 rpm to give (**5**) which was washed with DMF (3 × 100 mL), DCM (3 × 100 mL), and DMF (1 × 100 mL).

3-bromothiophene-2-carboxamido-Gly-Gly-Arg (6): After swelling with dry DCM (80 mL) for 30 min and washing with DMF (80 mL), (**5**) was treated with a solution of 5 % TFA in DCM (80 mL) for 30 min at rt to give the protected tripeptide. The reaction mixture was collected and the resin was washed with DCM (3 × 100 mL). The solution was concentrated and dried for 12 hr *in vacuo*. Finally, deprotection of the side chain group was carried out with 50 mL TFA:H_2O:TIS (95:2.5:2.5) for 2 hr. TFA was then removed by evaporation and the final product (**6**) was obtained by purification with HPLC (**Figure S1**) (t_R = 7.91 min).

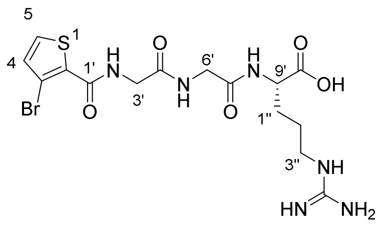

(6)

^{1}H NMR (400 MHz, DMSO-d6): δ 8.30 (td, J = 5.5, 2.6 Hz, 2H, NH), 8.08 (d, J = 7.8 Hz, 1H, CH(**5**)), 7.85 (d, J = 5.2 Hz, 1H, NH), 7.61 (d, J = 7.0 Hz, 1H, CH(**4**)), 7.22 (d, J = 5.2 Hz, 4H, NH), 4.19 (td, J = 8.2, 5.0 Hz, 1H, CH(**9'**)), 3.94 (d, J = 5.5 Hz, 2H, CH_2(**6'**)), 3.87 – 3.70 (m, 2H, CH_2(**3'**)), 3.08 (q, J = 6.7 Hz, 2H, CH_2(**3''**)), 1.74-1.59 (m, 2H, CH_2(**1''**)), 1.49 (qt, J = 7.4 Hz, 2H, CH_2(**2''**)). **^{13}C-NMR (100 MHz, $CDCl_3$):** δ 173.4, 168.6, 160.2, 156.7, 132.9, 132.0, 130.7, 124.0, 118.9, 110.1, 51.6, 42.7, 41.8, 28.3, 25.1. **IR (neat)**: 3330, 3190, 3100, 2970, 2930, 1630, 1530, 1415, 1245, 1200, 1135, 1085, 1050, 880, 760, 720, 685. **HRMS-ESI:** calculated for $C_{15}H_{22}BrN_6O_5S^+$: 477.0478; found for $C_{15}H_{22}BrN_6O_5S^+$: 477.0556.

a

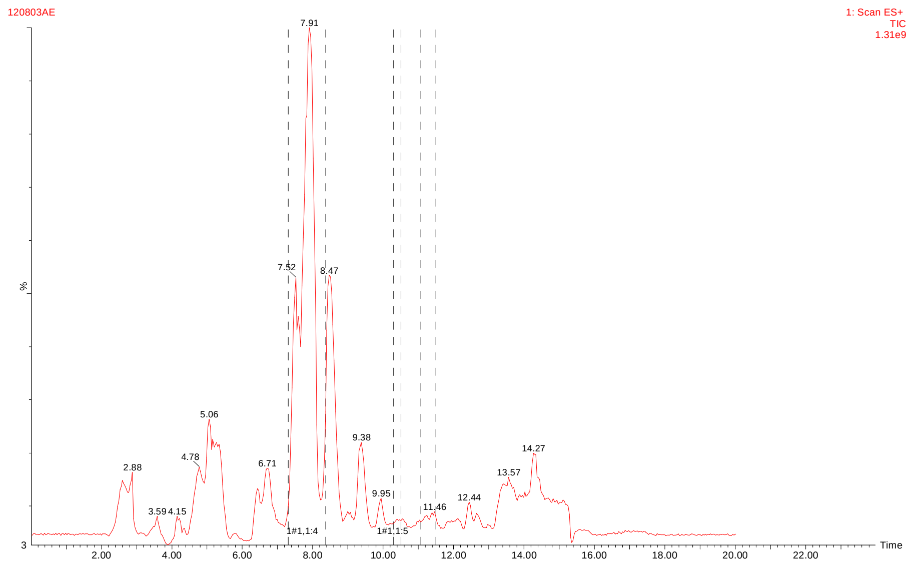
120803AE
1: Scan ES+
TIC
1.31e9
%
7.91
7.52
8.47
5.06
9.38
14.27
2.88
4.78
6.71
13.57
9.95
12.44
11.46
3.59
4.15
1#1,1:4
1#1,1:5
3
Time
2.00
4.00
6.00
8.00
10.00
12.00
14.00
16.00
18.00
20.00
22.00

b

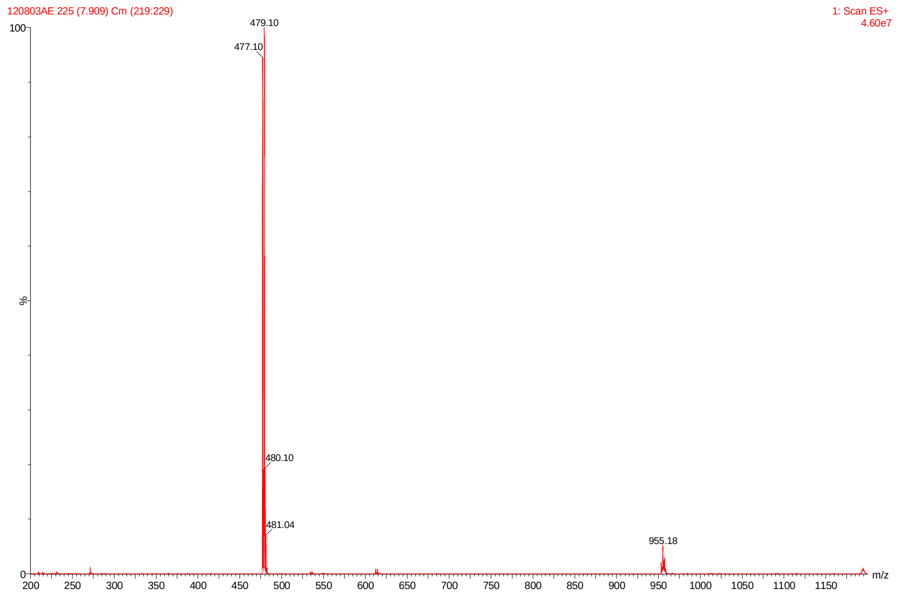
120803AE 225 (7.909) Cm (219:229)
1: Scan ES+
4.60e7
100
%
0
479.10
477.10
480.10
481.04
955.18
m/z
200
250
300
350
400
450
500
550
600
650
700
750
800
850
900
950
1000
1050
1100
1150

c

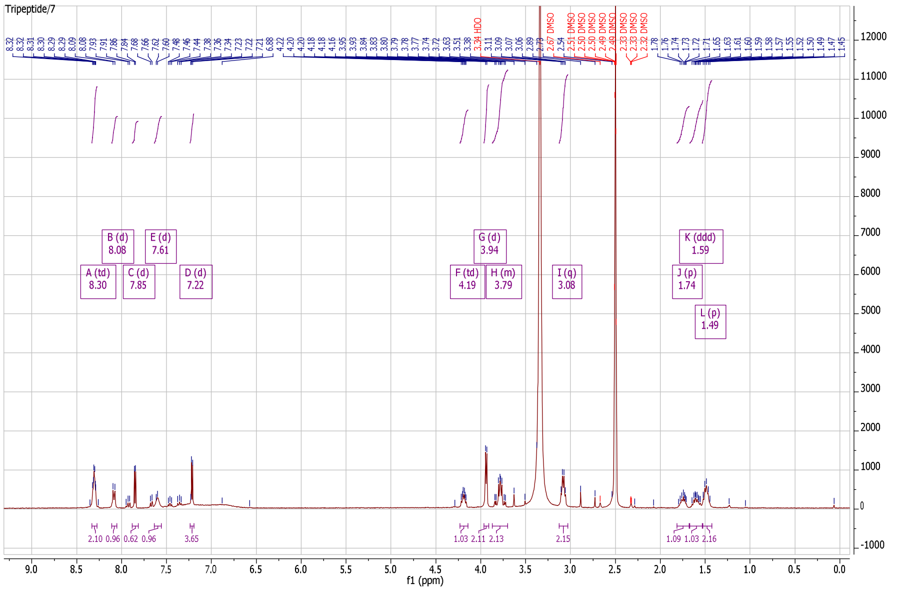

d

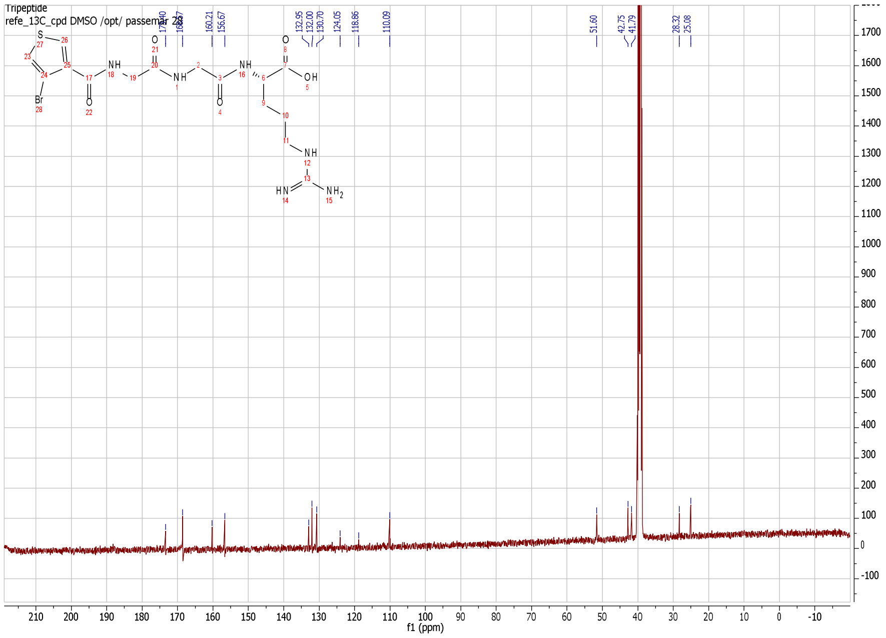

Figure S1 a): HPLC spectrum: 2 to 60 % of solvent B (ACN:HCOOH 99.9:0.1) in solvent A (H2O:HCOOH 99.9:0.1) in 10 min. **b)** LC-MS spectrum after HPLC purification. **c)** ^{1}H NMR of **6** in DMSO-d_6 at 400 MHz and 25° C. **d)** ^{13}C NMR of **6** in $CDCl_3$ at 100 MHz and 25° C.

Protein-Ligand Screening using Long-Lived Coherences (LLC) boosted by Dynamic Nuclear Polarization (DNP)

In the singlet-triplet basis set, Long-Lived Coherences (LLC) (1-3) can be defined as a linear combination of $|S_0\rangle\langle T_0|$ - $|T_0\rangle\langle S_0|$ and $|S_0\rangle\langle T_0|$ + $|T_0\rangle\langle S_0|$. These terms are equivalent to $I_x - S_x$ and $2I_yS_z - 2I_zS_y$ in the product basis. An LLC experiment comprises similar steps as required for the observation of Long-Lived States (LLS): excitation, sustaining and detection. First, the Boltzmann equilibrium population $I_z + S_z$ of an IS pair of chemically inequivalent spins is transformed into a density operator $I_x - S_x$ (Figure S2), or alternatively into $2I_yS_z - 2I_zS_y$. Then, during a variable interval τ_m, the two spins are rendered magnetically equivalent by applying a resonant *rf* field with a carrier ν_{rf} positioned at the average of the chemical shifts of the two spins that are involved in the LLC. After interrupting the *rf* irradiation, the free induction signal is acquired as usual. The detectable single-quantum coherences are best described in the product basis as a function of τ_m:

$$\sigma(\tau_m) = \left[(I_x - S_x)\cos(2\pi J_{IS}\tau_m) + \left(2I_yS_z - 2I_zS_y\right)\sin(2\pi J_{IS}\tau_m)\right]\exp\left(-\frac{\tau_m}{T_{LLC}}\right) \qquad \text{(S1)}$$

The observation of $I_x - S_x$ as function of τ_m gives an oscillatory decay that can be Fourier transformed to obtain a so-called *J*-spectrum with very narrow line widths $\Delta\nu_{LLC} = 1/(\pi T_{LLC})$. A LLC has a lifetime T_{LLC} that is often much longer than the conventional transverse relaxation time T_2 of the corresponding protons. Just like the lifetimes T_{LLS} of Long-Lived States, the lifetimes T_{LLC} can be dramatically reduced if the ligand that carries the LLC binds to a protein.

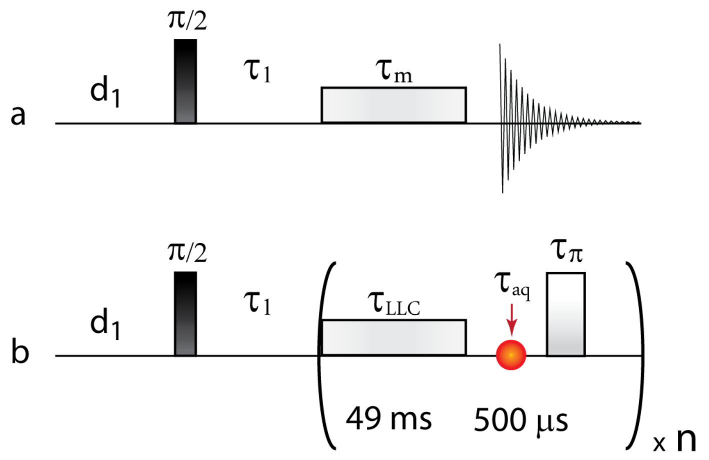

Figure S2: Pulse sequences designed to excite, sustain and detect LLC. (**a**) in the manner of two-dimensional spectroscopy, with N_1 steps $\tau_m = n_1\Delta\tau_m$, n_1 = 0, 1, 2, ..., (N_1-1), and (**b**) using a one-dimensional 'on-the-fly' fast acquisition method. Typically, $\tau_1 = 1/2\Delta\nu_{IS}$ to achieve an efficient conversion of $I_y + S_y$ into $I_x - S_x$ and τ_{aq} = 500 μs, $\Delta\tau_m$ = 50 ms to have a bandwidth of the *J*-spectrum of 20 Hz, τ_{aq} = 500 μs, $\tau_{LLC} + 2\tau_{aq} + \tau_\pi = \Delta\tau_m$ = 50 ms.

This oscillatory decay can be sampled by incrementing τ_m in the manner of two-dimensional spectroscopy (Fig. S2a). To reduce the experimental time, it is possible to sample the exponential decay of the sinusoidal signal by choosing delays τ_m in the vicinity of the maxima that appear at multiples of $1/J_{IS}$ (Fig.S 3). We have acquired signals for three τ_m delays that are close to each of these maxima. A local fit of these three signal amplitudes permits one to determine one of the maxima of

the sinusoidal signal (Fig.S 4). The value of T_{LLC} is obtained by a mono-exponential fit of the envelope of consecutive maxima (Fig S5).

By alternating intervals for signal observation and for sustaining the LLC, one can also observe N_1 points of an 'on-the-fly' LLC signal in a single shot (1) in the manner of one-dimensional spectroscopy (Fig. S2b). The *rf* irradiation is briefly interrupted so that part of the oscillating LLC is temporarily transformed into observable single-quantum coherence in a window $\tau_{aq,}$ refocused by a π pulse, before being transformed back into LLC by switching the *rf* irradiation on again. One of the advantages of this fast acquisition scheme is to make the LLC method fully compatible with dissolution DNP (4),

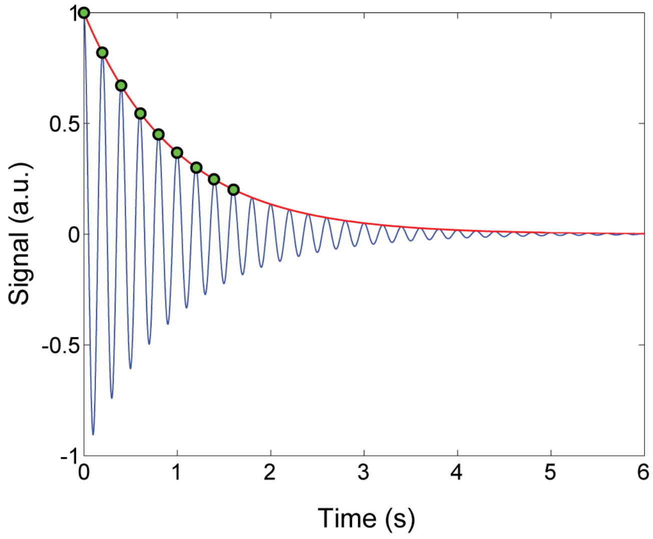

Figure S3: Sampling the oscillating LLC decay. Sampling around the maxima of the sinusoidally modulated decay of the LLC signals (green dots) allows one to determine the time constant T_{LLC} by fitting to an exponential curve (red).

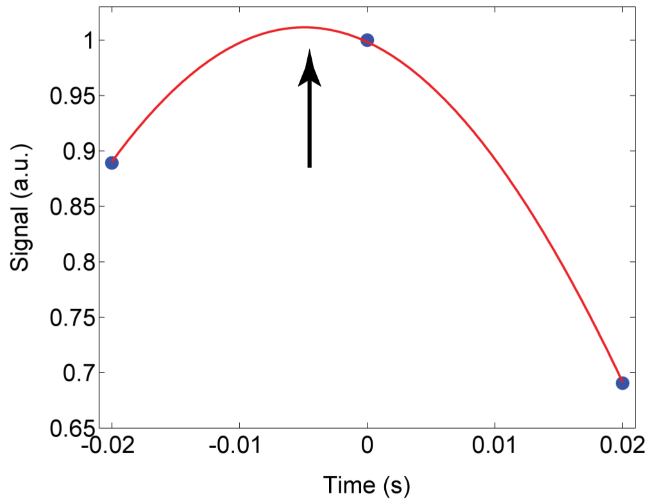

Figure S4: Local fit near three points. The signal amplitudes at three points (blue dots) taken near a maximum of the sinusoidally modulated decay of an LLC signal allows one to determine the amplitude of the maximum (arrow) by local fitting (red curve).

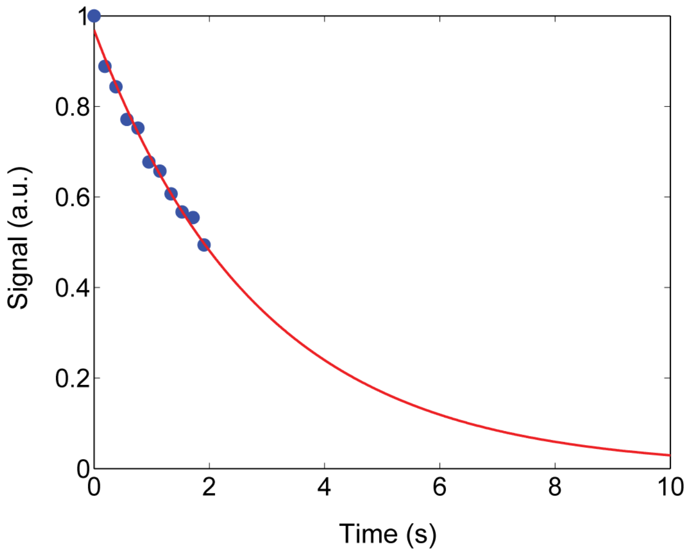

Figure S5: Experimental LLC decay. Fitting of 11 consecutive maxima of the sinusoidally modulated decay of an LLC signal allows one to determine T_{LLC}.

Since Long-Lived Coherences (LLC) belong to the class of zero-quantum coherences, their precession and decay are not sensitive to the inhomogeneity of the static magnetic field. If one excites an LLC *via* zero-quantum coherences, the efficiency of its excitation is not sensitive to the homogeneity of the static magnetic field (5). When all shim coil currents are set to zero (so that normal proton spectra show line widths of about 50 Hz), LLC spectra of free BT-GGR can be obtained with line widths as narrow as $\Delta\nu_{LLC}^{free}$ = 0.20 Hz (T_{LLC}^{free} = 1.5 s), *i.e.*, a resolution enhancement of a factor of 50 000 (5). As LLC spectra can be acquired in a single shot, the method can be combined with dissolution DNP (1, 4).

Although the lifetimes of Long-Lived Coherences (LLC) associated with the two diastereotopic protons of the central Glycine in GGR turned out to be rather disappointing, the LLC associated with the two aromatic protons of bromothiophene of BT-GGR (T_{LLC}^{free} = 1.5 s) is ideal for ligand screening. With a ligand/protein ratio $[L]_0/[P]_0$ = 40, a contrast C_{LLC} of 82 % was obtained. Figure 5c in the main text shows LLC spectra of 0.5 mM BT-GGR in the presence of 25 µM trypsin, either without competitor, or with 50 µM of the intermediate competitor apigenin, or with 50 µM of the stronger competitor myricetin. All LLC spectra were recorded "on the fly" with the sequence of Figure S2b. In the absence of competitor, the protein concentration available for binding with the "spy ligand" BT-GGR is $[P]_{free} = [P]_0$, so that one observes a fairly short T_{LLC}^{obs} = 0.81 s and LLC spectra with fairly broad lines $\Delta\nu_{LLC}^{obs} = 1/(\pi T_{LLC}^{obs})$ = 0.39 Hz. In the presence of a binder that has a stronger affinity for the protein than the "spy ligand" BT-GGR, $[P]_{free}$ decreases, T_{LLC}^{obs} becomes longer, the peak narrower, and its intensity higher (see Figure 5c and 5d of the main text).

References

1. Bornet A, Jannin S, Konter JA, Hautle P, van den Brandt B, & Bodenhausen G (2011) Ultra High-Resolution NMR: Sustained Induction Decays of Long-Lived Coherences. *J Am Chem Soc* 133(39):15644-15649.

2. Sarkar R, Ahuja P, Vasos PR, & Bodenhausen G (2010) Long-Lived Coherences for Homogeneous Line Narrowing in Spectroscopy. *Phys Rev Lett* 104(5):053001.
3. Sarkar R, Ahuja P, Vasos PR, Bornet A, Wagnieres O, & Bodenhausen G (2011) Long-lived coherences for line-narrowing in high-field NMR. *Prog Nucl Mag Res Sp* 59(1):83-90.
4. Ardenkjaer-Larsen JH, Fridlund B, Gram A, Hansson G, Hansson L, Lerche MH, Servin R, Thaning M, & Golman K (2003) Increase in signal-to-noise ratio of > 10,000 times in liquid-state NMR. *Proc. Natl Acad Sci USA* 100(18):10158-10163.
5. Chinthalapalli S, Bornet A, Segawa TF, Sarkar R, Jannin S, & Bodenhausen G (2012) Ultrahigh-Resolution Magnetic Resonance in Inhomogeneous Magnetic Fields: Two-Dimensional Long-Lived-Coherence Correlation Spectroscopy. *Phys Rev Lett* 109(4):047602.
